# Adenosine administration in supraventricular tachycardia

**DOI:** 10.1007/s12471-017-1033-9

**Published:** 2017-09-12

**Authors:** P. Robles Velasco, I. Monedero Sánchez, A. Rubio Caballero, M. Chichakli Cela, Y. González Doforno

**Affiliations:** Cardiology Unit, Hospital Universitario Fundación Alcorcon, Madrid, Spain

## Answer

**Fig. 1:**
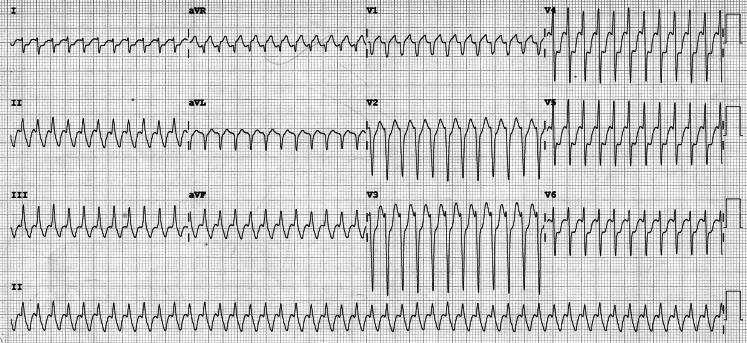
ECG after administration of adenosine. Atrial flutter with 3:1 atrioventricular conduction was initially observed for a few seconds, but it then changed to 1:1 atrioventricular conduction, showing the potential proarrhythmic effect of adenosine

The ECG during symptoms was strongly suggestive of typical atrial flutter. After adenosine administration, the heart rate slowed for a few seconds because of 3:1 atrioventricular (AV) conduction, but it then changed to 1:1 conduction response (Fig. 1). This was associated with haemodynamic instability, which required electrocardioversion. Although adenosine administration is usually innocuous, its potential harmful effects should not be underestimated. A careful analysis of the initial ECG could have foreseen this atypical response, as 1:1 AV conduction beats were observed [1]. These beats are indicators of a high sympathetic tone, which increased after adenosine administration, with the outcome described above [2]. This case shows the unusual but dangerous proarrhythmic effect of adenosine in patients with atrial flutter, after producing important sympathetic discharge and subsequent 1:1 AV conduction, as well as the presence of predictors in the ECG which discourage its administration [3].

## Conclusion

Atrial flutter with 1:1 atrioventricular conduction after administration of adenosine.
